# Cost-efficient filtration and replication strategies match or exceed detection sensitivity for marine eDNA diversity at an intertidal site under a standardized laboratory workflow

**DOI:** 10.1371/journal.pone.0350111

**Published:** 2026-09-10

**Authors:** Nicholas O. Schulte, Joseph M. Craine, Jessica Devitt, Galen W. Miller, Steven D. Gaines, Kevin D. Lafferty, Jessica M. Altstatt

**Affiliations:** 1 Jonah Ventures, Boulder, Colorado, United States of America; 2 Marine Science Institute, University of California, Santa Barbara, California, United States of America; 3 U.S. Geological Survey, Western Ecological Research Center, Santa Barbara, California, United States of America; Charles University: Univerzita Karlova, CZECHIA

## Abstract

Optimizing environmental DNA (eDNA) metabarcoding to maximize species detection while minimizing costs and effort remains a challenge, particularly in high-throughput workflows. In the marine intertidal zone off Point Conception, California, we compared eDNA richness accumulation between filtration approaches that differed in filter type and downstream processing, field and laboratory replicates, and independent and pooled polymerase chain reaction (PCR) replicates. Ten 10-cm^2^ capsule filters and 10 5-cm^2^ syringe disc filters were processed using a standardized laboratory workflow for 12S ribosomal ribonucleic acid (rRNA) (marine vertebrates) and 18S rRNA (metazoans and phytoplankton) metabarcoding, with 10 independent PCR replicates per filter. Because capsules generated larger lysate volumes than discs, the fixed extraction input volume under the standardized workflow resulted in less lysate being processed. A follow-up study pooled 10 PCR replicates for 12S rRNA. When comparing individual PCR replicates, individual filters, or all filters aggregated, discs detected greater marine vertebrate richness than capsules. Probabilistic models indicated that varying effective volume alone could produce similar richness accumulation patterns to those observed, though other filter characteristics were unaccounted for. Minimal differences in richness accumulation were detected between filters for 18S rRNA, and modeling indicated that this was not explained by higher initial copies of 18S rRNA. Meanwhile, vertebrate richness accumulation was similar when adding PCR replicates from the same sample or independent field samples across the short, relatively homogenous intertidal transect. Pooling PCR replicates had no detectable difference on richness compared to sequencing PCR replicates independently: a single pooled PCR replicate detected the same vertebrate richness as the sum of 10 independent PCR replicates. These results show that, under the standardized workflow and conditions tested here, eDNA programs may reduce costs and effort without detectable loss of richness by using filtration approaches aligned with extraction input and pooling technical replicates.

## Introduction

Environmental DNA (eDNA) metabarcoding has emerged as a tool for aquatic biodiversity monitoring, from freshwater streams and lakes to estuaries and coasts to coral reefs and open oceans [1]. With metabarcoding, the presence – if not relative abundance – of a wide variety of species can be measured. Target taxonomic groups can be selected based on the primers used, which range from general (e.g., all eukaryotes) to a relatively narrow subset of species (e.g., fishes) [2]. eDNA metabarcoding can also be a cost-effective, efficient, and less invasive alternative to traditional sampling techniques [3,4]. eDNA collection requires only minimal field equipment and training, and when coupled with the high throughput of independent analytical laboratories, surveys of aquatic eDNA become one of the most scalable tools for biodiversity monitoring across the tree of life. To meet this promise for large-scale biodiversity monitoring, however, eDNA methods must also scale so that thousands of samples can be collected and processed quickly, inexpensively, and accurately [5]. Optimizing methods requires even and repeatable comparisons of different techniques.

The costs to collect, process, and sequence a sample for eDNA are not insignificant. Thus, despite numerous successful surveys of aquatic biodiversity using eDNA [6–8], important questions remain about how best to maximize species detection with metabarcoding while minimizing costs and effort [9]. Optimizing eDNA metabarcoding requires reconciling methods [10,11] that vary in sampling design, filter characteristics, volumes of water filtered, DNA preservation, extraction protocols, primer choice, polymerase chain reaction (PCR) conditions, PCR replication, methods of indexing samples, post-PCR cleaning options, normalization approaches, size-selection for amplicons, sequencing platform, and sequencing depth [12,13]. The approaches used at each stage of the process are not independent and need to be considered holistically and in conjunction with environments sampled and the target biological groups [14–16]. In general, three variables are considered to strongly affect species detections: the volume of water filtered for a sample, the number of samples analyzed, and the sequencing depth used [17]. The general consensus is that the greater quantities of each variable, the greater the diversity detected – up to a given limit [18,19]. Yet, greater filtered water volume, sample number, and sequencing depth often come with higher costs, labor, and processing times [20], creating uncertainty about optimal approaches for eDNA analyses at scale.

A basic, but nuanced element of aquatic eDNA analysis is the water filter and its downstream processing constraints, such as the volume of lysate generated for DNA extraction. To filter large volumes of water for a single sample (e.g., 1–60 L), filters with membrane surface areas ≥ 10-cm^2^ are often used. These can range in price from US$15 – $150 per unit [21]. Independent of the relatively high material cost and length of filtering time, high surface area filters often generate large volumes of lysate (≥ 2 mL) from which DNA is extracted [22]. The large amounts of lysate must be either subsampled, which reduces the effective proportion of sample volume filtered (assuming DNA is well mixed in the lysate), or DNA must be concentrated prior to extraction, which adds to the processing time, labor, and cost of laboratory materials [23,24]. In contrast, small-diameter syringe filters typically process less water because of smaller surface areas (e.g., 5-cm^2^ for a 25 mm diameter filter) but are generally lower-cost (<US$1/ unit) and generate less lysate (< 1 mL), which can more easily be extracted in full under the common extraction constraint of fixed input volumes [25]. How these linked differences in filtration and processing influence eDNA detection when laboratory extraction effort is held constant is not well understood.

Similar to the benefits of filtering more water, processing more field samples – either as site replicates or single samples from multiple sites – typically yields greater diversity [26]. Water samples are also usually taken in replicates to increase the total water volume filtered and to estimate error. In aquatic systems that are considered to have high spatial heterogeneity of DNA of different species, multiple field samples are thought to be needed to fully characterize the regional species pool [27,28]. In contrast, if DNA is relatively well-mixed in the environment, fewer samples may be necessary – especially if each is more thoroughly analyzed [29,30].

An alternative way to increase the effective volume of water that is sampled is to process a sample more exhaustively in the laboratory. This is possible because little (< 5%) of the DNA extract is used for a single PCR reaction, meaning that DNA molecules for every species in the assemblage may not be abundant enough to be present and amplify in each subsample [31]. Stochastic subsampling and potential amplification stochasticity during PCR can result in detecting different species from PCR replicates of the same sample [32,33]. In these cases, species richness may accumulate with additional PCR replicates from a single sample just as it can with PCR reactions from independent samples. Since PCR replicates from within the same sample do not require additional sampling consumables, collection effort, or extraction, a more thorough analysis of a single field sample in the laboratory may reduce time and costs by only requiring a single DNA extraction compared to processing multiple field samples superficially. PCR replicates can also be pooled before sequencing to further reduce costs [34]. However, few studies have considered trade-offs in species detection between independent PCR replicates and pooled PCR samples, and pooling replicates has the disadvantage of not generating estimates of variance.

Given outstanding questions about the most efficient ways to maximize species detection, we explored how filtration approaches and sample replication strategies affected cost, effort, and species detection along a rocky intertidal coastline. To assess filtration approaches, we compared the ability of 5-cm^2^ disc and 10-cm^2^ capsule filters to detect marine vertebrates and eukaryotes (metazoans and phytoplankton) while applying equal laboratory effort in extracting DNA from each. We then compared richness detected from additional field samples versus separate PCR replicates versus pooled PCR replicates. By collecting multiple field samples and sequencing multiple PCR replicates independently, we generated effort-response curves that allowed for equal-effort comparisons of richness for both filtration approaches. Once the general patterns of richness accumulation with additional sampling and laboratory effort were determined, we explored whether these general patterns could be reproduced by modeling random draws of species from pools that differed in overall species abundance, evenness, and richness. We then addressed whether pooling first-round PCR products of independent PCR replicates was as effective for detecting species as sequencing each PCR replicate separately. Before and after rarefying PCR replicate read data so that all 10 replicates combined had the same sequencing depth as each of two pooled replicates, we compared richness between the 10 replicates and pooled replicates for each field sample. In addition to comparing richness detection from each approach, we consider their respective costs and effort.

## Methods

Water samples were collected from 10 intertidal sites spaced approximately 15 m apart and between 0.3–0.6 m tidal height along the rocky shoreline of Government Point within the Point Conception State Marine Reserve, California (34.44243472, −120.4534482). The Government Point coastline is predominantly consolidated bedrock and is the site of long-term monitoring and intertidal biodiversity surveys as part of the Multi-Agency Rocky Intertidal Network, which target barnacles, mussels, brown macroalgae, seagrass, and sea stars, among other taxa [35]. The main purpose of our sampling effort was to evaluate a cost-effective sampling design for eDNA-based biodiversity monitoring of metazoans (particularly fishes) and phytoplankton along the Pacific coastline of the United States.

At each site, approximately 5 L of water was collected from the shoreline on 31 January 2024 in a dual-port polyethylene bag with one port for water collection and a second for subsequent water filtration (“Baleen bag” from Jonah Ventures, Boulder, CO, USA) (S1 Table). Bags of water were stored on ice in a cooler for up to 9 hours prior to processing in the laboratory. For each site, two samples were collected via vacuum filtration using a pump and a different filter type per sample. For the first sample, bags were shaken, and 1000 mL of water was passed through a 0.45 µm pore size, 10-cm^2^ surface area polyvinylidene fluoride (PVDF) membrane Millipore Sterivex capsule filter (MilliporeSigma, Burlington, MA, USA; Cat. # SVHVB1010). Then, for the second sample, bags were shaken to resuspend any particulate matter, and water was passed through a 1.0 µm pore size, 25-mm diameter nylon membrane syringe disc filter until it clogged, which was estimated as the point when flow declined to one drop (~0.05 mL) per second filtered. Filter types were selected based on their widespread use in marine eDNA workflows, and their comparison reflects two commonly implemented filtration approaches rather than an attempt to isolate all potential filtering factors such as membrane material or pore size.

On average, 653 mL of water were passed through the disc filters, with a range of 500–900 mL. For both capsules and discs, filtration time took approximately 10–30 minutes per sample. 1000 mL of filtered tap water was filtered through each of three capsule filters, and 500 mL of tap water was filtered through one disc filter for a total of four field blanks. After filtration, air was pulled through the filter to purge remnant water from the housing. Longmire’s buffer, which contains 0.5% of the anionic detergent sodium dodecyl sulfate (SDS), was then added to the filter housing as a preservative in volumes based on the housing capacity: 2 mL for capsules and 0.4 mL for discs. Filters were stored in the refrigerator (4 °C) for 2 days prior to shipment at ambient temperature to Jonah Ventures laboratory in Boulder, Colorado. Samples were then stored overnight in a refrigerator prior to DNA extraction the following day. For a second round of sampling on 29 May 2024, 10 nearby sites were visited, and an average of 539 mL was passed through disc filters only and preserved, stored, and shipped the same way as the previous sampling campaign (S2 Table). 600 mL of filtered tap water was filtered through each of three disc filters as field blanks, which were then processed the same as field samples.

Prior to DNA extraction, sample filters with Longmire’s preservative were heated to 56 °C for at least one hour. The heat and time allow the anionic detergent in Longmire’s to solubilize organic debris collected on the filters. Sample preservative was then transferred to sterile tubes under a laminar flow hood. For capsule filters, using a 5-mL syringe of ~2.5 mL of air, all preservative buffer (~2 mL) was pushed through the filter into a 5-mL tube containing 50 µL proteinase K. Proteinase K is an enzyme that digests proteins, thus releasing otherwise bound DNA. For disc filters, 500 µL of Omega Bio-tek tissue lysis (TL) buffer (Omega Bio-tek, Norcross, GA, USA; Cat. # PD061, a proprietary solution that contains 1–5% anionic detergent) mixed with 50 µL of proteinase K was heated in a 3-mL syringe and was pushed into the filter housing followed by ~2.5 mL of air. TL buffer was added to the disc filter extraction as part of a standardized high-throughput workflow to generate sufficient lysate volume for subsequent extraction. All lysate (containing the sample preservative and lysis buffer, ~ 0.95 mL) was collected in a 2-mL tube. For both filtration approaches, tubes containing all lysate were incubated at 56 °C overnight to allow for proteinase K digestion before continuing extraction the next day. Before extraction, lysate was vortexed, centrifuged, and – for capsule filter lysate – transferred into 2-mL tubes. For each sample, 700 µL of lysate was used for extraction. For capsule filters, this constituted ~34% of the total lysate; for discs, ~ 74%. DNA from the lysate was extracted using the Omega Bio-tek Mag-Bind Blood & Tissue DNA HDQ 96 Kit (4x96 Preps) (Cat. # M6399-01). Extractions were carried out on a STARlet platform running Venus 4 (Hamilton Company, Reno, NV, USA), using a method developed by the extraction kit manufacturer and adapted as needed. After extraction, DNA was eluted into 100 µL elution buffer and stored at −20 °C. Two extraction blanks were run in conjunction with the January 2024 sample extractions; however, no extraction blanks were run alongside the May 2024 samples. No contamination was detected in the January extraction blanks.

From each genomic DNA sample, hypervariable regions of the mitochondrial 12S ribosomal ribonucleic acid (rRNA) and nuclear 18S V9 rRNA genes were amplified by PCR. The 12S rRNA primers were MiFishU–F (5’-GTCGGTAAAACTCGTGCCAGC-3’) and MiFishU-R (5’-CATAGTGGGGTATCTAATCCCAGTTTG-3’) with spacer regions [36]. The 18S rRNA primers were 1389F (5’-TTGTACACACCGCCC-3’) and 1510R (5’-CCTTCYGCAGGTTCACCTAC-3’) [37]. Forward and reverse primers also contained a 5’ adaptor sequence to allow for subsequent indexing and Illumina sequencing. For 12S rRNA, 10 replicate aliquots of extracted DNA per sample were then amplified via PCR; for 18S rRNA, only one aliquot per sample was amplified due to budget constraints.

For amplification of the 12S rRNA primer set, 12.5 µL of Qiagen Multiplex PCR kit (1000) (Qiagen, Hilden, Germany; Cat.# 206145) master mix was used. 0.5 µL of each primer (10 mM), 3.0 µL of gDNA, 5 µL of the included “Q-Solution”, and 8.5 µL of nuclease-free water was added to the master mix for a total reaction volume of 30 µL. For amplification of the 18S rRNA primer set, 12.5 µL of Promega PCR Master Mix (Promega, Madison, WI, USA; Cat. # M5133) was used. 0.5 µL of each primer (10 mM), 3.0 µL of gDNA, and 8.5 µL of nuclease-free water was added to the master mix for a total reaction volume of 25 µL. DNA was PCR amplified using the following conditions: for 12S rRNA, initial denaturation at 95 °C for 3 minutes, followed by 45 cycles of 20 seconds at 98 °C, 30 seconds at 60 °C, and 30 seconds at 72 °C, and a final elongation at 72 °C for 10 minutes. For 18S rRNA, initial denaturation at 94 °C for 3 minutes, followed by 30 cycles of 30 seconds at 94°C, 1 minute at 57 °C, 90 seconds at 72 °C, and a final elongation at 72 °C for 10 minutes. One no template control (NTC) was run as a negative control for 12S rRNA alongside the January 2024 samples and showed no detectable amplification. No NTCs were run for 18S rRNA or for 12S rRNA alongside the May 2024 samples. The absence of these controls limits the ability to evaluate laboratory-derived contamination in these samples, particularly for low-level detections. Amplicons were cleaned by incubating the reactions with Exo1/SAP for 30 minutes at 37 °C followed by inactivation at 95 °C for 5 minutes and storage at −20 °C. For each field sample from the May 2024 sampling, two pooled replicates for 12S rRNA were created in addition to the 10 independent (unpooled) PCR replicates. Each pooled replicate was prepared by combining 3 µL aliquots from each of the 10 first round 12S rRNA independent PCR replicates, resulting in a total volume of 30 µL per pooled replicate. These pooled replicates were treated as separate samples during the second round of PCR.

A second round of PCR was performed to complete the sequencing library constructs, appending the final Illumina sequencing adapters and integrating dual 10-nucleotide index sequences for the forward and reverse primers. The indexing PCR included Promega Master mix, 0.5 µM of each primer and 2 µl of template DNA (cleaned amplicon from the first PCR reaction) and consisted of an initial denaturation of 95 °C for 3 minutes followed by 8 cycles of 95 °C for 30 sec, 55 °C for 30 seconds, and 72 °C for 30 seconds.

Final indexed amplicons from each sample were cleaned and normalized using a magnetic bead-based protocol based on [38] with minor alterations. A 15 µL aliquot of PCR amplicon was purified and normalized using Cytiva SpeedBead magnetic carboxylate modified particles (Cytiva, Marlborough, MA, USA; Cat. # 45152105050250). Samples were then pooled together by adding 5 µL of each normalized sample to the pool. Sample library pools were sent for sequencing on an Illumina NovaSeq 6000 at the Texas A&M Agrilife Genomics and Bioinformatics Sequencing Core facility using the SP Reagent Kit v1.5 (500 cycles) (Illumina, San Diego, CA, USA; Cat. # 20028402).

Raw sequence data were demultiplexed using *pheniqs* v2.1.0 [39], enforcing strict matching of sample barcode indices (i.e., no errors). *Cutadapt* v3.4 [40] was then used to remove gene primers from the forward and reverse reads, discarding any read pairs where one or both primers (including a 6-bp, fully degenerate prefix for 12S rRNA) were not found at the expected location (5’) with an error rate < 0.15. Read pairs were then merged using *vsearch* v2.15.2 [41], discarding resulting sequences with a length of < 130 bp or > 210 bp (12S rRNA) or < 87 bp or > 186 bp (18S rRNA), or with a maximum expected error rate > 0.5 bp. For each sample, exact sequence variants (ESVs) were generated using the ‘unoise3’ denoising algorithm as implemented in *vsearch* [42], using an alpha value of 5 and discarding unique raw sequences observed less than 8 times. Counts of the resulting ESVs were then compiled and putative chimeras were removed using the ‘uchime3’ algorithm, as implemented in *vsearch*.

For each final ESV, a consensus taxonomy was assigned using a custom best-hits algorithm and a reference database consisting of publicly available sequences (GenBank for 12S rRNA and SILVA for 18S rRNA) as well as Jonah Ventures voucher sequences records. Reference database searching used an exhaustive semi-global pairwise alignment with *vsearch*, and match quality was quantified using a custom, query-centric approach, where the percent match ignores terminal gaps in the target sequence, but not the query sequence. The consensus taxonomy was then generated using either all 100% matching reference sequences or all reference sequences within 1% of the top match, accepting the reference taxonomy for any taxonomic level with > 90% agreement across the top hits.

To assign consistent species-level taxonomy to ESVs, consensus taxonomy was adjusted based on observations and reference hits. First, species-level taxonomy was retained for all ESVs with ≥ 95% match and a consensus species assignment. For other ESVs, all reference hits within 1% match to the most similar reference (i.e., top match) were examined. If a hit species was present in observed consensus species, a pairwise alignment was generated, and the ESV was assigned the species name if within 2% similarity to an ESV with consensus taxonomy. Otherwise, a provisional species name was assigned based on unique species of the top hits. Read counts of ESVs with the same consensus species taxonomy were then summed. Although consensus species are effectively operational taxonomic units (OTUs) based on top reference hit matches and ESV similarity, we refer to them as “species” throughout the text, since they represent taxa that were determined according to approximately species-level taxonomy and sequence similarity. However, all species names should be considered provisional given potentially incomplete reference databases, lack of distribution-informed classification, and uncertainty in assignment to species ranks. We do not assume that the species names assigned to ESVs always correctly indicate the species from which the sequence came. Provisional species taxonomy, representative sequences, and read counts for each gene region and sampling event are available in S3–S5 Tables.

All statistical analyses were performed in R v.4.3.0 [43]. First, reads from nontarget taxa were removed, retaining only marine vertebrates (bony fishes, cartilaginous fishes, and marine mammals) for 12S rRNA and marine algae and metazoans for 18S rRNA (i.e., excluding archaea, bacteria, fungi, and heterotrophic protists). No 12S rRNA ESVs were detected in the three field blanks, two extraction blanks, or the NTC that were run alongside January samples, nor were any ESVs detected in the three field blanks run alongside May samples. Therefore, all 12S rRNA ESVs and their original read counts were retained. For 18S rRNA, no ESVs were detected in the two extraction blanks. One of the four field blanks contained 24 18S rRNA ESVs, 16 of which were also detected in field samples. However, no single ESV in the field blank exceeded 132 reads, and no field sample contained more than 13 of the 24 ESVs found in the field blank. Given the absence of dominant shared ESVs, low read counts in blanks, and no evidence of systematic cross-contamination patterns, all 18S rRNA ESVs and their original read counts were retained. Nevertheless, NTCs were not included for 18S rRNA or the 12S rRNA May campaign, so the absence of controls for these samples limits assessment of potential contamination.

To measure the distribution of high and low abundance species across samples, cumulative rank abundance curves were calculated by summing reads for each species across samples and dividing them by the total reads. Logarithmic regressions were calculated by transforming the species rank index using ‘lm’ in *stats*. To estimate the total observed and predicted species pools across PCR replicates and field samples, species accumulation was calculated using ‘estimateD’ and ‘iNEXT’ in *iNEXT* v.2.0.20 [44] and ‘specaccum’ in *vegan* v.2.6-2 [45]. For analysis of pooled replicates, to standardize sequencing effort between individual PCR replicates and pooled PCR replicates for a given sample, individual PCR replicates were proportionally rarefied using ‘rrarefy’ in *vegan* so that all 10 replicates combined summed to the lowest sequencing depth of the two pooled replicates. Finally, to measure compositional differences between filtration approaches and among field samples, non-metric multidimensional scaling (NMDS) ordinations were performed on presence/absence-based Jaccard dissimilarity using ‘metaMDS’ in *vegan*.

Species accumulation curves were simulated using simple probabilistic models to explore potential mechanisms underlying the following patterns observed in the field study: (Q_1_) larger species pools in discs than capsules across 10 12S rRNA samples, (Q_2_) higher predicted asymptotes in discs than capsules for 12S rRNA, and (Q_3_) smaller differences between discs and capsules in 18S rRNA species accumulation than 12S rRNA. Our associated hypotheses were: (H_1_) larger effective volumes of water filtered are associated with larger species pools, as observed in disc filters; (H_2_) larger effective volumes of water filtered result in detecting the full species pool in fewer samples than smaller volumes; and (H_3_) high DNA copies/ L result in the same magnitude and rate of species accumulation across effective volumes of water filtered.

Probabilistic species accumulation models included the number of PCR replicates (*n*_*replicates*_), species pool size (*n*_*species*_), volume of water in L containing *n*_*species*_ (*n*_*volume*_), number of DNA copies per L (*n*_*copies*_ *= n*_*species*_*/ n*_*volume*_), proportion of copies filtered (*proportion*_*copies*_), and the skewness of DNA copies across species (*n*_*skew*_). *n*_*replicates*_ was set at 10 for Q_1_ and Q_3_ and at 10, 100, 1000, and 10000 for Q_2_. *n*_*species*_ was set at either 90 (the number of observed 12S rRNA species across all samples, for Q_1-2_) or 1500 (the rounded asymptote for all 18S rRNA samples, for Q_3_). *n*_*volume*_ was set based on results from species accumulation curves: 70 L for Q_1-2_ (the number of samples predicted for asymptotic diversity from 10 12S rRNA PCR replicates for both discs and capsules, assuming 1 L water/ sample) and 100 L for Q_3_ (the number of samples predicted for asymptotic diversity from all 18S rRNA samples, assuming 1 L water/ sample). To represent a range across expected low (12S rRNA) and high (18S rRNA) initial DNA template assemblages, *n*_*copies*_ was set at 1000 copies/ L for Q_1-2_ and 1000, 10000, and 100000 copies/ L for Q_3_. Copy number per species was randomly calculated based on an overall right-skewed distribution (i.e., few abundant species) of *n*_*species*_ across *n*_*volume*_. The skewness of the distribution was determined by running simulations with skewness values ranging from 0.1 to 5 and selecting the skewness that yielded accumulation most similar to that observed across 10 PCR replicates for 12S rRNA (skewness = 3.5). *proportion*_*copies*_ was defined from 0.1 to 1 at 0.1 increments to simulate the effective volume of water filtered in L as the result of water volume filtered * proportion of lysate extracted. For capsule filters, the observed effective volume of water filtered was 0.34 (proportion of lysate extracted) * 1 (L of water filtered in the field study) = 0.34 L and for discs, 0.70 * 0.65 (average L water filtered through discs in the field study) = 0.46 L. For each of 10 simulations per combination of model parameters, species copy numbers were randomly sampled based on *n*_*copies*_ * *proportion*_*copies*_ * *proportion*_*DNA*_ (a constant, the proportion of extracted DNA used in PCR: 3 μL of 100 μL, or 0.03) and repeated *n*_*replicates*_ times. The final *n*_*replicates*_ sampled assemblages were used to calculate observed and predicted species accumulation curves with standard deviation using *iNEXT*.

Capsule filters most closely followed the simulated accumulation slope of an effective volume of approximately 0.175 L, whereas disc filters had a greater slope than any simulated volume but shared a starting species pool with 0.5 L and a final species pool with 0.7 L. These modeled volumes differed from the empirically calculated effective volumes: lower than expected for capsules (empirically calculated effective volume of 0.34 L) and higher than expected for discs (0.46 L), indicating that effective volume alone did not fully reproduce the observed patterns and that additional factors such as pore size, membrane material, or extraction efficiency may also contribute. Subsequent simulations addressing Q_2_ and Q_3_ were designed to explore how variation in effective volume influences the number of samples required to approach asymptotic richness and the effect of DNA concentration on accumulation patterns, rather than to validate the empirical calculations. Therefore, for these simulations we used the modeled effective volumes that most closely approximated the observed accumulation slopes and ranges (0.175 L for capsules and 0.6 L for discs) as calibrated representations of workflow-level effective sampling effort. These values therefore serve as fitted approximations for exploratory modeling, not as direct empirical measurements.

## Results

### Patterns of marine vertebrates from 12S rRNA

When comparing the 12S rRNA species pools of 100 PCR replicates (10 field samples * 10 PCR replicates) for both disc and capsule filtration approaches from the first sampling campaign, a total of 78 marine vertebrate species were detected for discs, as opposed to just 49 for capsules. Between the two approaches, a total of 85 species were detected. Of these, 77 were Actinopteri (bony fishes), 2 were Chondrichthyes (cartilaginous fishes, only in discs), and 5 were marine mammals, constituting 99.865%, 0.002%, and 0.133% of total reads, respectively. Actinopteri included taxa common in near shore (sculpin, surfperch), kelp forest and rocky reef (rockfishes, kelp bass), and open water (sardine, mackerel) habitats. The Chondrichthyes species were both bottom-dwelling sharks, *Cephaloscyllium ventriosum* (swell shark) and *Heterodontus francisci* (horn shark). The marine mammals were the locally common *Eschrichtius robustus* (gray whale), *Tursiops truncatus* (bottlenose dolphin), *Enhydra lutris* (sea otter), *Zalophus californianus* (California sea lion), and *Phoca vitulina* (harbor seal).

The greater richness for disc filters compared to capsule filters was also observed when comparing just a single PCR replicate for a given sample or all 10 PCR replicates for individual samples. Comparing filtration approaches for just one PCR replicate, 9.7 ± 0.5 marine vertebrate species were detected, on average, for disc filters, while capsule filters averaged 4.1 ± 0.3 species (*p* < 0.001) (Table 1). When including all 10 PCR replicates for an individual filter, 31.0 ± 1.9 marine vertebrate species were detected for disc filters, on average. Capsule filters averaged 46% fewer marine vertebrate species across 10 PCR replicates than the disc filters (17.0 ± 1.9 species, *p* < 0.001).

**Table 1:** Summary statistics of reads and richness for each primer set and filtration approach from the first sampling event in January 2024 (mean ± standard error).

	12S rRNA		18S rRNA	
	Disc	Capsule	Disc	Capsule
Mean filtered reads per PCR replicate (x1000)	80.6 ± 6.5	48.7 ± 6.1	106.1 ± 6.4	118.9 ± 7.0
Mean species per PCR replicate	9.7 ± 0.5	4.1 ± 0.3	381.0 ± 14.4	338.9 ± 17.4
Mean species per field sample (10 PCR replicates)	31.0 ± 1.9	17.0 ± 1.9	n/a	n/a
Total species observed	78	49	933	910
Total species constituting 95% abundance	20	20	167	170
Number species detected in just 1 field sample	34	21	364	378
Number species detected in just 1 PCR replicate	32	21	364	378
Number species detected in fewer than 10 PCR replicates	55	37	n/a	n/a
Total species predicted from accumulation	134 ± 45	85 ± 46	1447 ± 118	1450 ± 101
Total PCR replicates predicted to be required for 95% of asymptotic richness	475	472	62	62
Total PCR replicates predicted to be required to detect asymptotic richness	898	784	113	107

**Fig 1:**
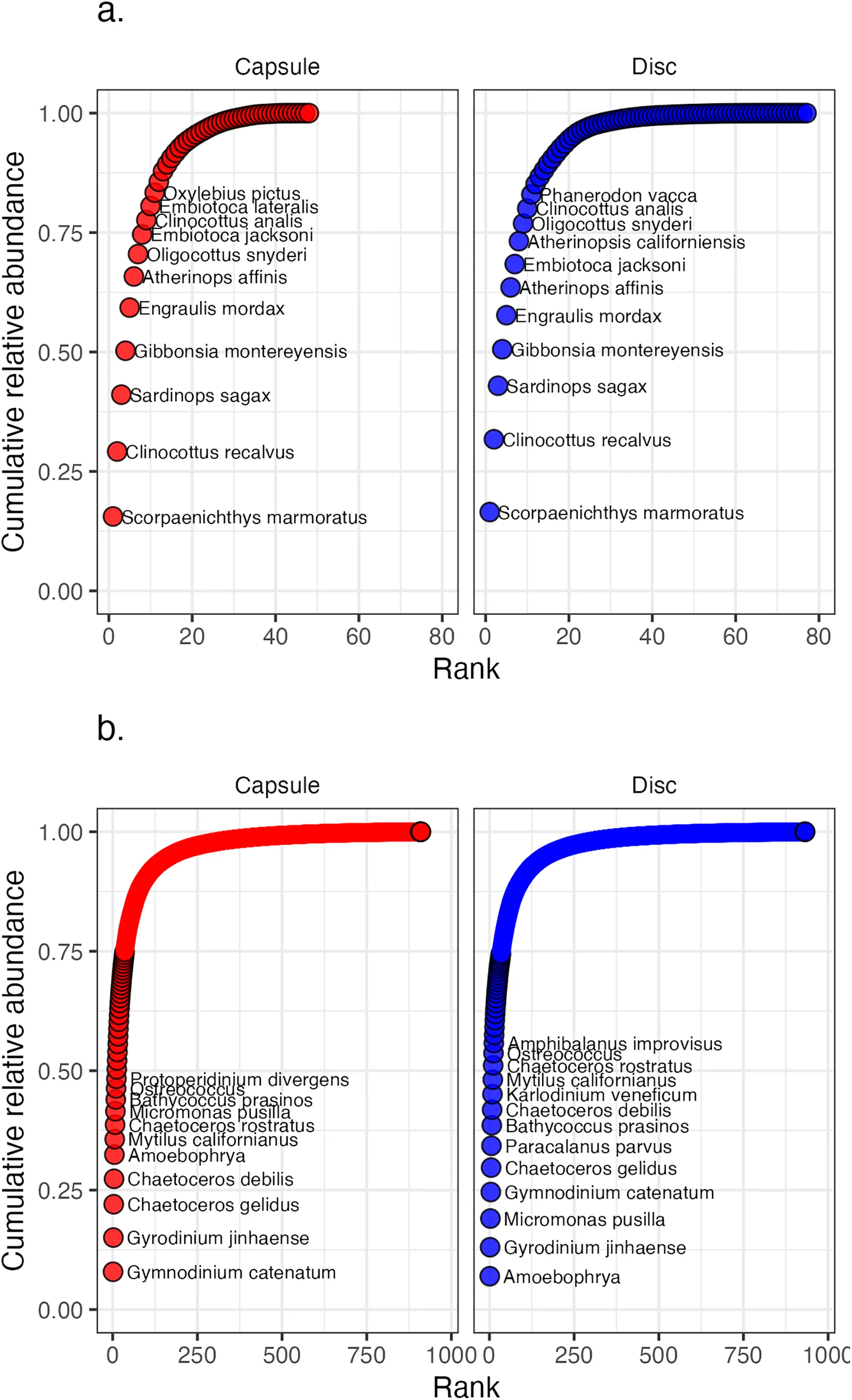
Cumulative rank relative abundance of species across PCR replicates for (a) 12S rRNA and (b) 18S rRNA. Each point is a species at its given rank.

**Fig 2:**
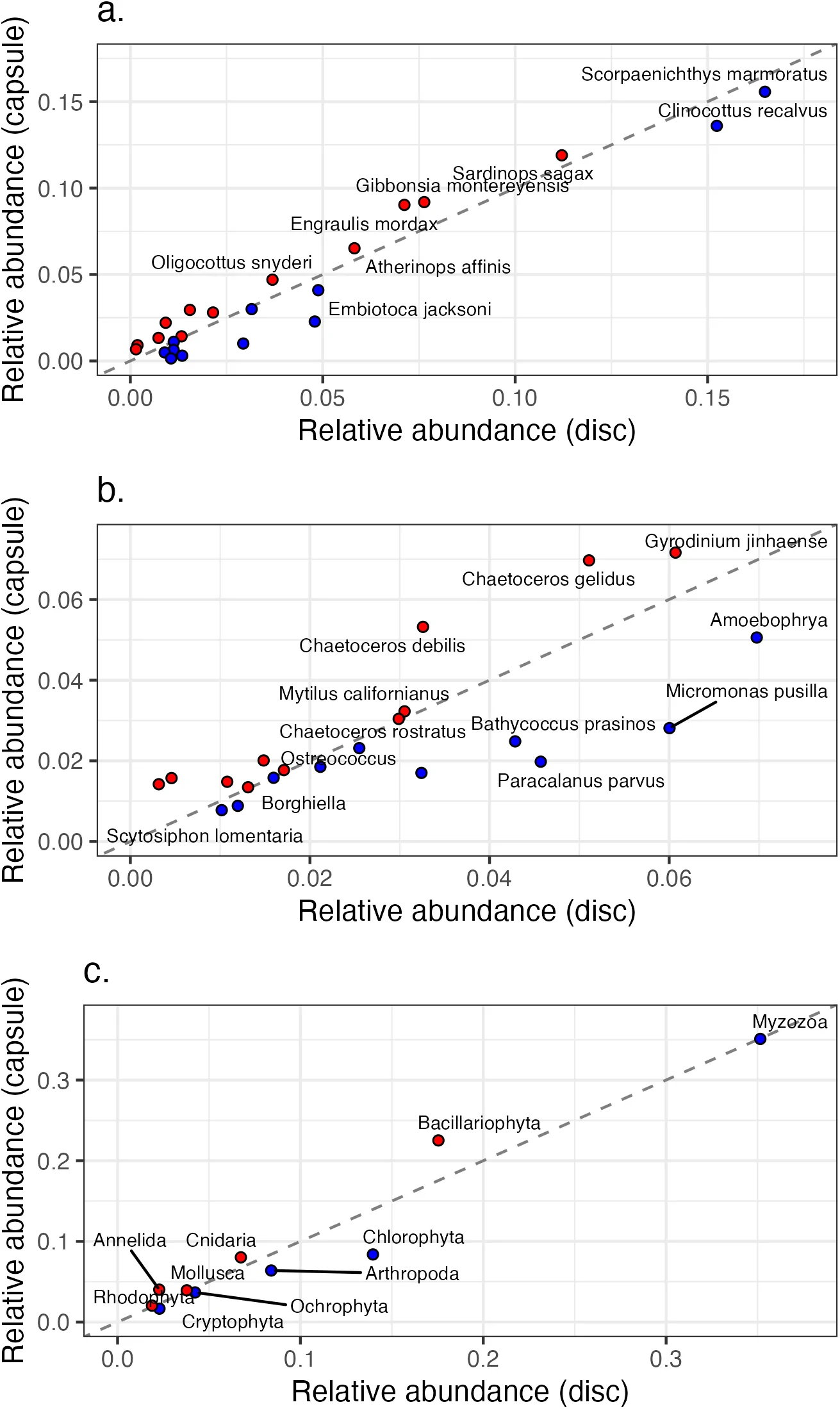
Comparison of species relative abundances across PCR replicates between disc and capsule filtration approaches for the (a) 20 most abundant 12S rRNA species (three species were not shared between filtration approaches), (b) 20 most abundant18S rRNA species (two species were not shared between filtration approaches), and (c) 10 most abundant 18S rRNA phyla. Blue fill denotes species with higher relative abundance in discs; red fill for higher relative abundance in capsules. Dashed = 1:1.

**Fig 3:**
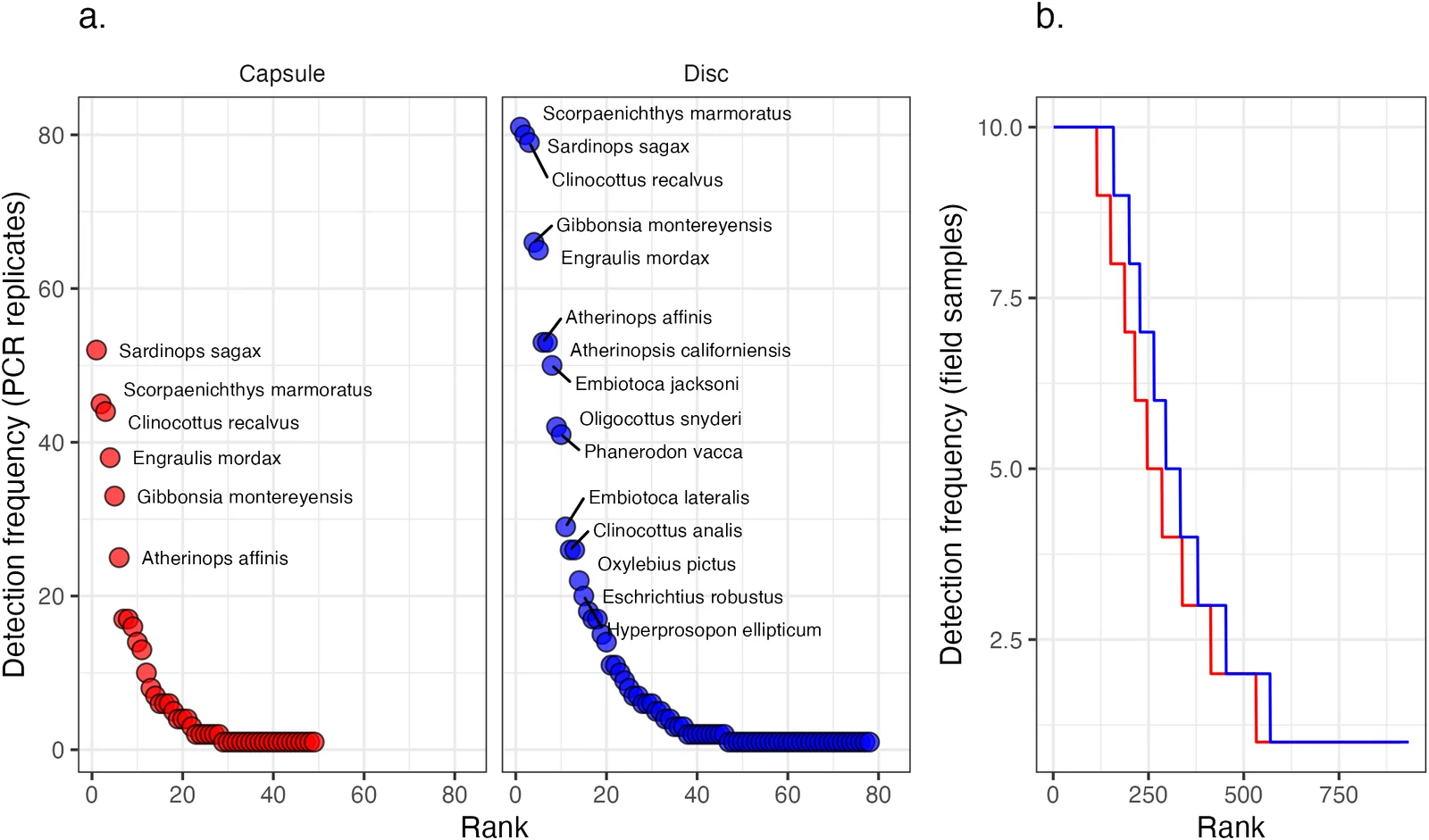
Detection frequency of each species by filtration approach. For (a) 12S rRNA, species present in at least 20 PCR replicates are labeled. For (b) 18S rRNA, too many species occur in each number of samples to label.

For both disc and capsule filters, cumulative rank abundance conformed to lognormal distributions (R^2^ = 0.84 and 0.91, respectively) (Fig 1a). Just 20 species constituted 95% of the reads for each filtration approach, 17 of which were shared between disc and capsule filters (Fig 2a). In general, vertebrate species that were highly abundant from disc filters were also highly abundant from capsule filters (Pearson r = 0.97, *p* < 0.001; Fig 2a). The five most abundant species (based on total reads) for both filtration approaches were fish species common to the waters off Point Conception: *Scorpaenichthys marmoratus* (cabezon), *Clinocottus recalvus* (bald sculpin), *Sardinops sagax* (Pacific sardine), *Gibbonsia montereyensis* (crevice kelpfish), and *Engraulis mordax* (northern anchovy). These 5 species accounted for 58% and 60% of the reads for disc and capsule filters, respectively. The greater total richness detected with disc filters relative to capsule filters was from relatively low abundance species. The additional 29 species found in disc filters constituted just 0.22% of the total reads for marine vertebrates. Examining patterns of prevalence, the top 5 fish species were found, on average, in 74% of the PCR replicates for discs and 42% for capsule filters. For discs, a total of 32 species were found in just one PCR replicate as opposed to 21 species for capsule filters (Fig 3a).

**Fig 4:**
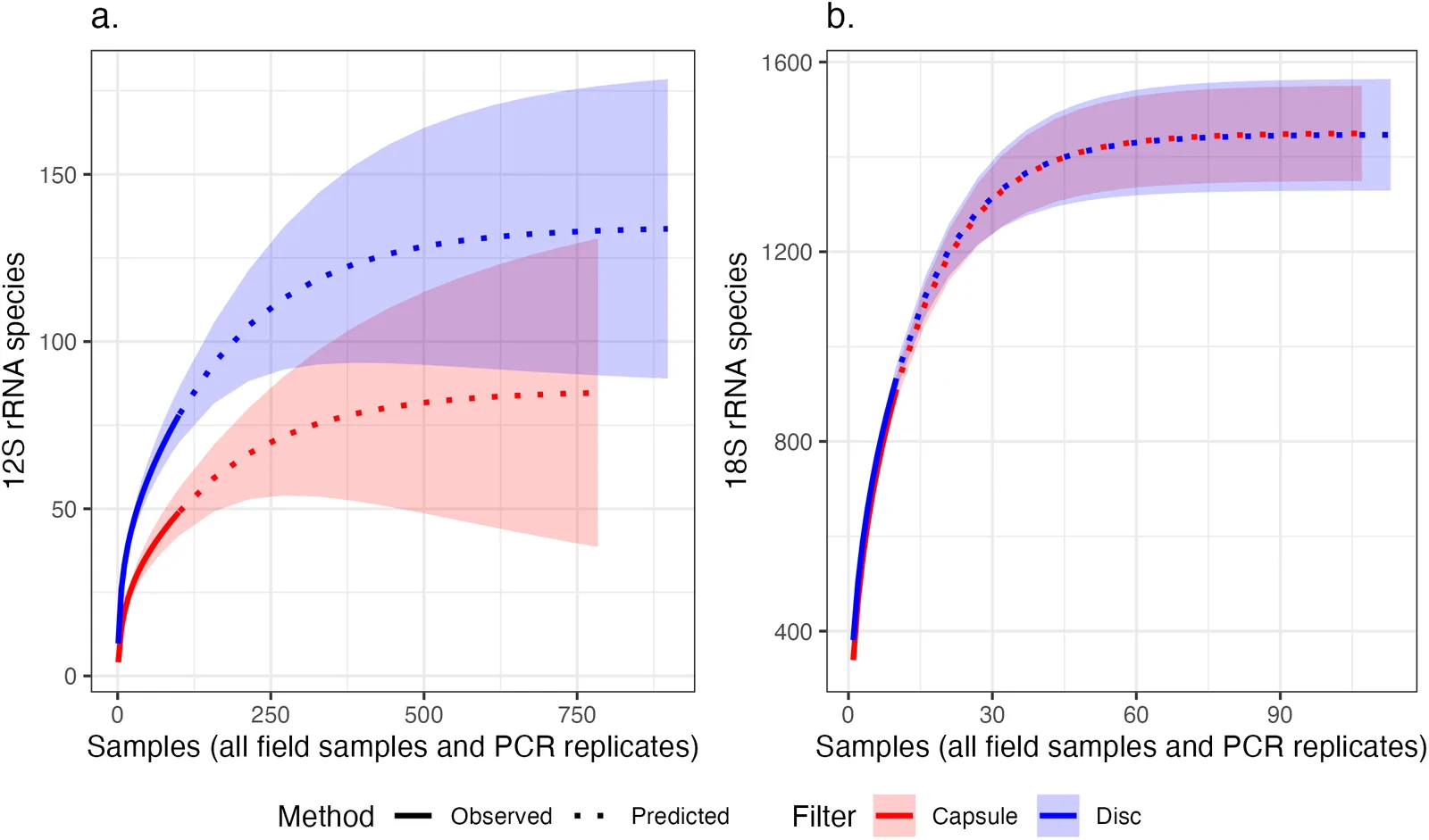
Observed and predicted richness accumulation across (a) all 12S rRNA samples (10 PCR replicates for each of 10 field samples) and (b) all 18S rRNA samples (one PCR replicate for each of 10 field samples).

**Fig 5:**
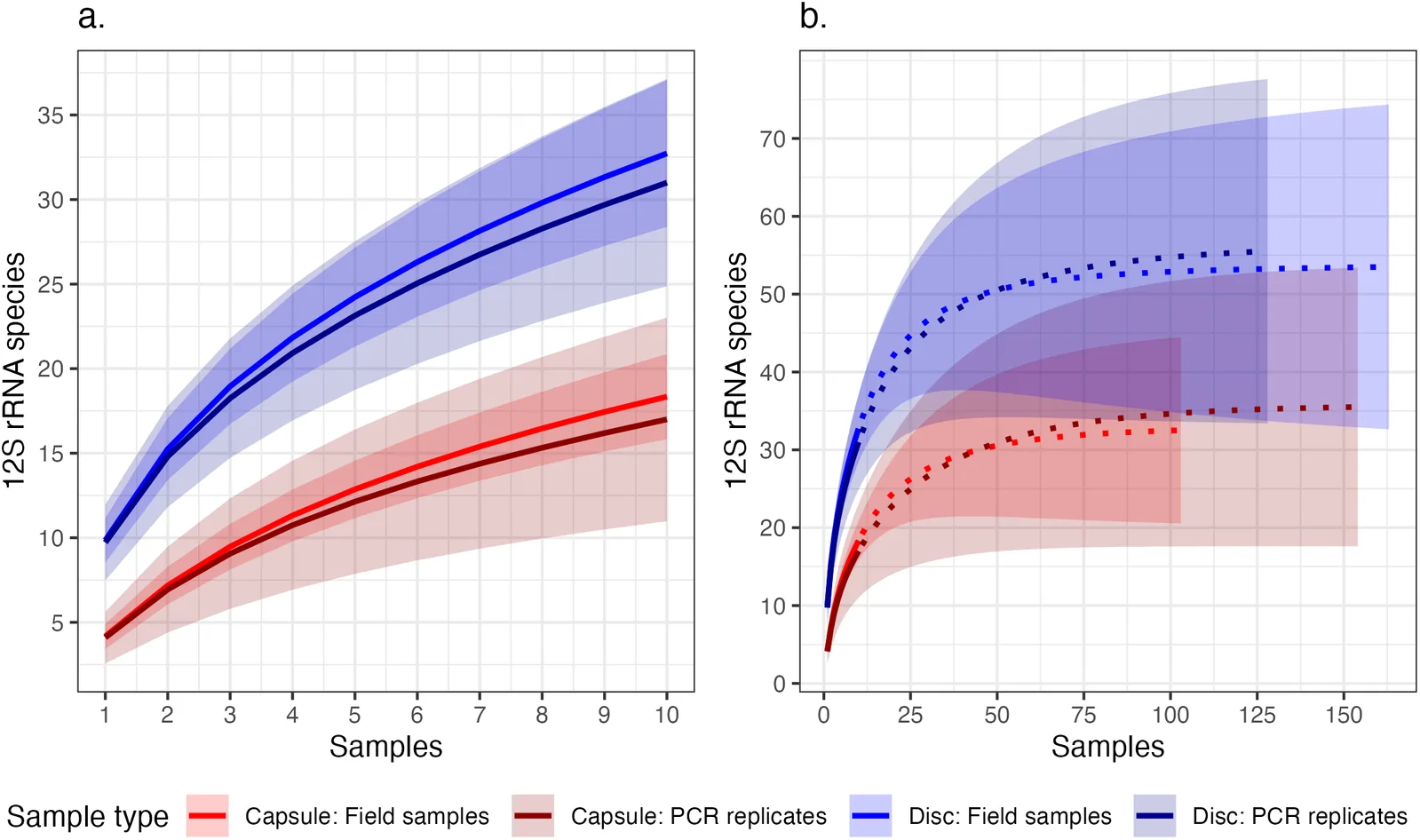
12S rRNA richness accumulation across field samples (mean of 99 random draws of one PCR replicate per field sample) and PCR replicates (mean of each of 10 field samples) for (a) 10 observed samples and (b) observed and predicted samples to richness asymptote.

Due to a high proportion of rare taxa, observed marine vertebrate richness for each filtration approach was less than 60% of that predicted by extrapolating from accumulation curves generated across all samples (Fig 4a), 10 PCR replicates per field sample (Fig 5), and one PCR replicate for each of 10 field samples (Fig 5). Extrapolating from the 100 PCR replicates (10 PCR replicates for each of 10 field samples), disc filters were predicted to detect 134 ± 45 marine vertebrate species, and detecting maximum richness would require 898 PCR replicates (Table 1). Capsule filters were predicted to detect 85 ± 46 species, requiring 784 PCR replicates.

Neither accumulation rate nor predicted richness was greater if PCR replicates were drawn from independent field samples than if they were drawn from a single field sample (Fig 5). For disc filters, the average richness drawn from 10 PCR replicates from the same field sample was 31 ± 6, while the average richness drawn from one PCR replicate for each of the 10 field samples (99 random draws) was 33 ± 4. Asymptotic richness would be predicted to be 56 ± 34 if one could pull all replicates from the same field sample or 54 ± 32 if all replicates were pulled from independent field samples similar to the 10 samples of this experiment. For capsule filters, the average richness was 17 ± 6 for PCR replicates and 18 ± 3 for field samples. Asymptotic richness was 36 ± 18 for same-sample PCR replicates and 33 ± 21 species for PCR replicates pulled from field replicates.

**Fig 6:**
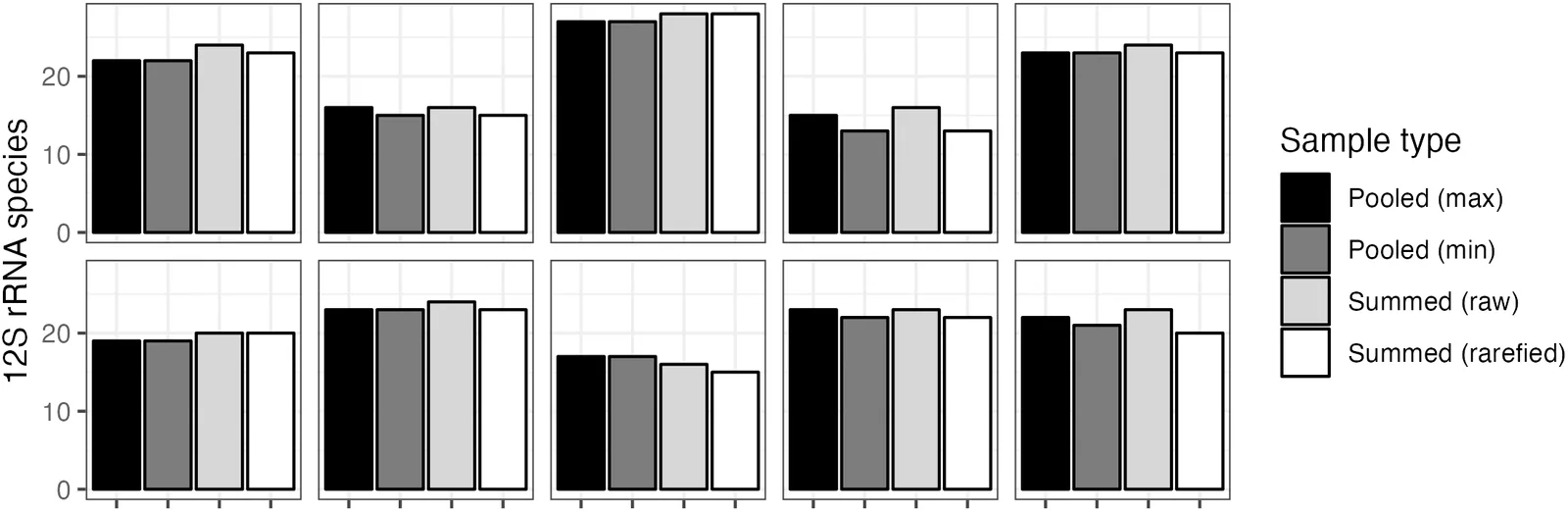
12S rRNA richness from each pooled sample (two per field sample) and from aggregating across 10 independent PCR replicates before (Summed [raw]) and after (Summed [rarefied]) rarefying sequencing depth to that of the pooled samples. Each panel is a separate field sample.

Pooled samples yielded similar richness as summing PCR replicate reads with or without controlling for differences in sequencing depth, based on samples from May 2024 (Fig 6). Prior to rarefaction, sequencing depth of independent PCR replicates averaged 14607 ± 569 filtered reads and sequencing depth of pooled samples averaged 17131 ± 1481 filtered reads. Across the 10 field samples, vertebrate richness averaged 20.5 ± 1.3 for each pooled sample. For each field sample, richness for all 10 independent PCR replicates summed together averaged 21.4 ± 1.3 before rarefying and 20.0 ± 1.4 after rarefying. Richness was no different between the two pooled samples for 6 of the 10 field samples, differed by one species for 3 samples, and differed by two species for only 1 sample. Meanwhile, a single pooled sample detected twice the richness on average as a single independent PCR replicate from the same field sample.

**Fig 7:**
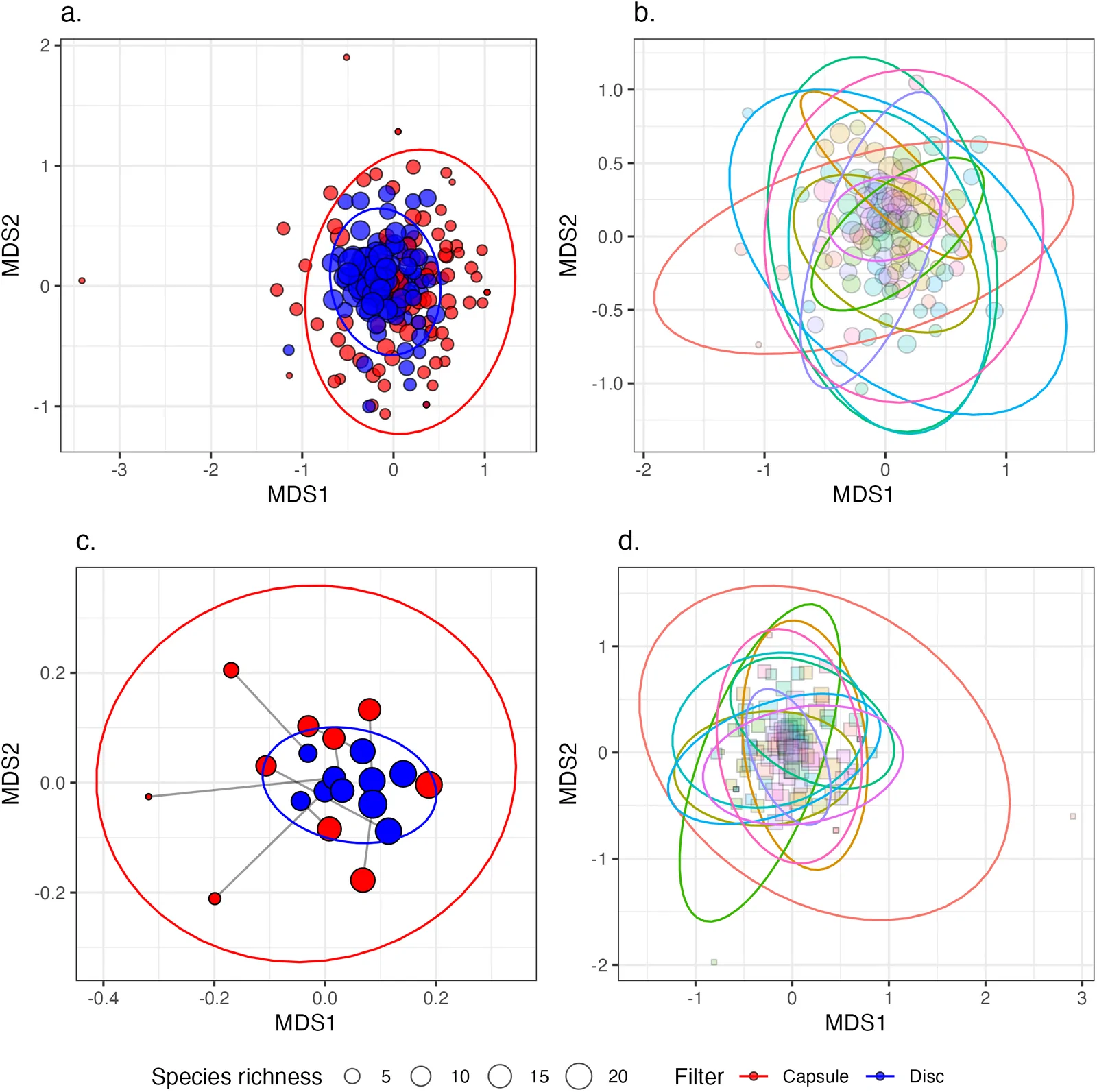
Non-metric multidimensional scaling (NMDS) ordination plots from Jaccard dissimilarity (presence/absence) for: (a) all 12S rRNA samples by filter, (b) 12S rRNA disc filters by site, (c) all 18S rRNA samples by filter (lines connect disc and capsule filters for the same site), and (d) 12S rRNA capsule filters by site. Ellipses represent 95% confidence intervals of group variation.

Based on ordinations of Jaccard presence/absence dissimilarity from the January 2024 sampling, marine vertebrate community composition was largely similar across samples when comparing the two filtration approaches (Fig 7a,b,d). Community composition showed a statistically detectable difference between filtration approaches (PERMANOVA, R^2^ = 0.04, *p* = 0.001), but this difference was small and primarily driven by variation in dispersion among samples (test of homogeneity of dispersion, *p* = 0.001), with little difference in group centroids (centroid permutation test, *p* = 0.029). For example, the difference in centroids along NMDS Axis 1 was only 4.0% of the range of Axis 1 values, and the difference in centroids along Axis 2 was only 1.3% of the Axis 2 range. Furthermore, the 95% confidence interval ellipse around disc samples was entirely nested within that of capsule samples.

Examining the pattern of community composition across sites, there was little indication of community composition differing among sites with either filtration approach. Of 45 pairs of field samples, only 3 differed significantly for capsule filters (pairwise PERMANOVA, R^2^ ≤ 0.17, adjusted *p* < 0.05). No field samples differed significantly in community composition or centroids among disc filters (pairwise PERMANOVA, R^2^ ≤ 0.13, adjusted *p* > 0.090).

### Patterns of marine eukaryotes from 18S rRNA

Across all 10 field samples, disc filters detected 933 metazoan and phytoplankton species across 30 phyla, and capsule filters detected 910 species across 31 phyla (Table 1). For both filtration approaches, the five most abundant phyla were Myzozoa (dinoflagellates, 35% of reads each), Bacillariophyta (diatoms, 18% and 23% of reads for discs and capsules, respectively), Chlorophyta (green algae, 14% and 8%), Arthropoda (8% and 6%), and Cnidaria (8% and 7%) (Fig 2c).

When compared at equivalent laboratory effort, disc filters yielded similar eukaryotic richness as capsule filters. With just one PCR replicate per field sample, richness averaged 381.0 ± 14.4 for disc filters. Richness per capsule filter was not significantly different than with discs, averaging 338.9 ± 17.4 (*p* = 0.08) (Table 1).

For both filtration approaches, the four most abundant species constituted nearly 25% of the total reads (Fig 1b-c). The most abundant taxa in the disc filters were *Amoebophrya* sp. (a parasitic dinoflagellate), *Gyrodinium jinhaense* (a heterotrophic dinoflagellate), *Micromonas pusilla* (a picoplanktic green alga), and *Gymnodinium catenatum* (a chain-forming, potentially toxigenic dinoflagellate). For capsule filters, the four most abundant taxa also included *Gymnodinium catenatum* and *Gyrodinium jinhaense*, as well as *Chaetoceros gelidus* and *C*. *debilis* (both colonial, planktic diatoms). Rank abundance curves for both discs and capsules closely followed a lognormal distribution (R^2^ = 0.78 for each).

In general, eukaryote species and phyla that were highly abundant from disc filters were also highly abundant from capsule filters (by species: *r* = 0.76, *p* < 0.001; by phyla: *r* = 0.97, *p* < 0.001; Fig 2b-c). Notably, *Amoebophrya* sp., *Micromonas pusilla*, *Paracalanus parvus* (a cosmopolitan copepod), and *Bathycoccus prasinos* (a picoplanktic green alga) were relatively more abundant with discs than capsules. Meanwhile, *Gyrodinium jinhaense*, *Chaetoceros gelidus*, and *C. debilis* were relatively more abundant with capsules.

Filtration approach did not strongly affect cumulative 18S rRNA richness. The observed marine algal and metazoan richness in the 10 samples was 65% and 63% of that predicted by extrapolating from accumulation curves generated across all samples for disc and capsule filters, respectively (Fig 4b). Extrapolating from the 10 field samples, the asymptotic richness with disc filters was predicted to be 1447 ± 118. Detecting maximum richness with a single PCR replicate per sample would require 113 field samples (Table 1). Asymptotic richness with capsule filters was predicted to be 1450 ± 101, requiring 107 field samples with a single PCR replicate.

Based on ordinations of Jaccard dissimilarity from presence/absence (Fig 7c), filtration approach showed a statistically detectable but small difference in 18S rRNA community composition across field samples (PERMANOVA, R^2^ = 0.06, *p* = 0.032), which was primarily driven by differences in dispersion among samples (test of homogeneity of dispersion, *p* = 0.001), with no detectable difference in centroids (centroid permutation test, *p* = 0.151). As with 12S rRNA, the 95% confidence interval ellipse around disc samples was entirely nested within that of capsule samples for 18S rRNA.

### Modeling

**Fig 8:**
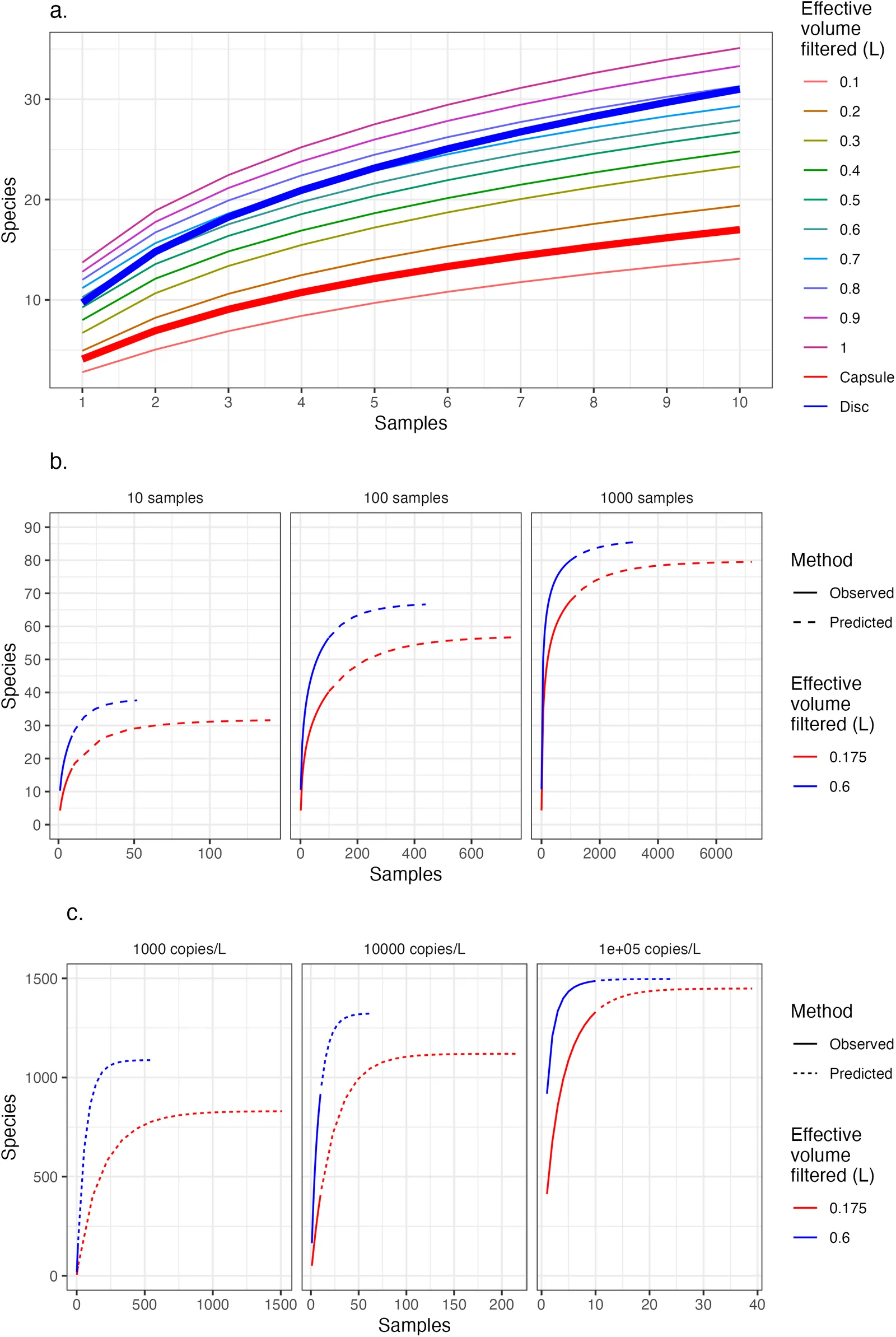
Results from accumulation simulations. (a) Accumulation across 10 samples varying effective volume filtered to simulate 12S rRNA patterns. Bold lines are observed accumulation from 12S rRNA. (b) Accumulation across a range of samples (panels) for effective volumes identified in (a). The simulated species pool is 90 species. (c) Accumulation across a range of DNA copies/ L (panels) across 10 samples and predictions for effective volumes identified in (a) to simulate 18S rRNA patterns. The simulated species pool is 1500 species.

Exploratory modeling indicated that differences in effective volume of water processed alone (i.e., volume of water filtered in the field * proportion of lysate extracted) produced broadly similar – but not identical – richness accumulation patterns to the higher cumulative 12S rRNA richness observed in disc filters relative to capsule filters. Simulated accumulation curves reproduced the general shape and magnitude of the observed patterns, but with modeled effective volumes that differed from observed (Fig 8). Increasing either the number of PCR replicates or the effective volume filtered was associated with increasing richness (Fig 8a). For 10 PCR replicates with parameters tuned to approximate the observed 12S rRNA data, increasing effective volume in 0.1 L increments yielded approximately five additional species between 0.1 L and 0.3 L, and roughly two additional species per 0.1 L above 0.3 L (Fig 8a). Simulated accumulation curves at 0.175 L and 0.6 L most closely approximated the observed slopes for capsules and discs, respectively, reproducing the empirically observed richness after 10 replicates of 17 species for capsules and 31 species for discs. However, effective volume alone did not fully capture the empirical values, as the modeled effective volume most similar to capsule accumulation (0.175 L) was 50% lower than the empirically calculated 0.34 L, and the modeled effective volume most similar to disc accumulation (0.6 L) was 33% higher than the empirically calculated 0.46 L.

To investigate whether predicted asymptotic richness for capsule and disc filters would converge after repeated sampling, we simulated and predicted species accumulation to 1000 samples using the modeled effective volumes that best approximated observed accumulation (0.175 L for capsules and 0.6 L for discs). Predicted differences in asymptotes between the 0.175 L samples and 0.6 L samples were maintained across 10, 100, and 1000 PCR replicates (Fig 8b). Neither 10, 100, nor 1000 PCR replicates reached the known species pool of 90 for either effective volume.

Increasing the number of DNA copies in an initial volume of water was not sufficient to entirely reproduce the observed high similarity in richness in disc and capsule filter accumulation for the 18S rRNA samples (Fig 8c). Instead, simulated accumulation for the 0.175 L samples (representing capsule filters) was slower (i.e., had a lower slope) than that for 0.6 L samples (disc filters) across the range of eDNA concentrations tested. Increasing eDNA concentration increased both initial and asymptotic richness but did not substantially change the relative accumulation rates between volumes.

## Discussion

Filtration and sample replication strategies for eDNA analysis of marine metazoan and phytoplankton diversity from a rocky intertidal site varied in their putative costs, effort, and detection under a standardized laboratory workflow. Yet, the expected tradeoff between cost and effort was not apparent in our results. The lower-cost filtration approach of processing small disc filters was as good or better at detecting marine vertebrate richness than processing larger, more expensive capsule filters, and modeling indicated that this may be driven in part by how completely the filter lysate was processed. Likewise, lower-cost pooled technical PCR replicates were as effective at detecting richness as more expensive independent technical replicates or even more expensive independent field samples taken at 15-m intervals.

Most eDNA methods comparisons focus on maximizing yield rather than efficiency. Furthermore, effort across disparate filter types and extraction methods (e.g., whole filters versus filter lysate versus direct water centrifugation) may not be able to be controlled. For example, a previous study [23] targeted maximum yield from 41 filtration-by-extraction treatments, each of which varied in laboratory handling, processing time, and material cost. DNA extraction protocol is known to affect eDNA yield [46,47], and the optimal method may vary with study-specific environmental conditions [32,48,49]. In one case, [50] found that a commercial DNA extraction kit with inhibitor removal outperformed similar cost extraction kits lacking inhibitor removal in turbid water, while the lower-cost phenol-chloroform-isoamyl alcohol extractions were the most time consuming and sensitive to inhibitors. Therefore, when not controlling for equal laboratory conditions and effort, conclusions about DNA yield or diversity cannot be attributed to any one filtration approach, specific processing step(s), and/or the amount of laboratory effort. For example, another study [19] compared water precipitation, 17.4-cm^2^ open disc filters, and 600-cm^2^ capsule filters and found high similarity between disc and capsule filter species detection and DNA yield. Yet, the disparate extraction methods used add uncertainty as to the extent to which water volume, filter characteristics, and/or extraction method each controlled the observed similarity.

In our study, filtration approaches differed in filter characteristics and lysis conditions, but comparisons were made under a standardized extraction input constraint to represent high-throughput, scalable workflows. Laboratory effort was controlled by using the same volume of filter lysate in DNA extractions for both filtration approaches. Under this constraint, less than 40% of capsule lysate was extracted compared to nearly 75% from discs. The disc filter approach detected nearly twice as many marine vertebrate (12S rRNA) species as capsule filters within and across samples, despite having half the surface area, a larger pore size, and filtering 35% less water on average. Empirically, this corresponded to effective volumes of 0.34 L for capsule filters and 0.46 L for disc filters after accounting for water volume filtered and lysate proportion extracted. Simulations indicated that varying effective volume alone was sufficient to generate broadly similar, though not identical, species accumulation patterns to those observed between filters, suggesting that effective volume is an important contributor to detecting richness.

Discrepancies between modeled and empirical patterns indicate that additional factors such as differences in membrane material, pore size, or lysis buffer chemistry between workflows may also contribute to observed differences in detection. Beyond richness, disc and capsule filtration approaches detected largely overlapping vertebrate assemblages, with small but statistically detectable compositional differences driven by greater dispersion among capsule filters rather than shifts in average composition, indicating that differences were primarily due to rare taxa, which are likely a constitutive feature of low-template environmental samples. Taken together, these results suggest that processing lower-cost disc filters (<US$1 each) can be as or more effective at measuring marine eDNA vertebrate diversity at our intertidal site than processing higher-cost capsule filters (> US$15 each) when laboratory effort is equal.

Nevertheless, our results do not mean that 25-mm syringe disc filters will always capture greater diversity than capsule filters with larger surface areas. Rather, to obtain the same effective volume of a disc filter, more water must be passed through a capsule filter given the lysate volume extraction constraint, or a greater proportion of the lysate needs to be analyzed. Indeed, several studies have shown that, holding filter type and all laboratory processes constant, increasing the actual water volume filtered resulted in higher richness [30], more DNA copies [51], and higher detection rates [52] from eDNA. In our study, accumulation curves indicate that at least 400 mL more water (1400 mL total) would need to be filtered per capsule filter to reach the mean effective volume filtered by disc filters, which is similar to the 1300 mL estimated from simple mathematical scaling that would need to be filtered to be proportional to the amount of water filtered by a disc filter with half the surface area of capsule filters. For capsule filters in our study, filtering 400 mL of additional water would require at least 10 minutes of additional filtering per sample, though filtration rates would likely slow as the filter becomes more clogged. For processing many samples at once, filtering more water per sample would require a substantial increase in hands-on time (e.g., an additional 3 + hours per 20 filters processed in the present study assuming all samples would be processed in series).

With our method of extraction that used a single loading step of 700 µL of filter lysate followed by a two-step magnetic bead binding step, we extracted less than 40% of capsule lysate versus nearly 75% from discs. Validated methods are available to extract greater amounts of lysate within a single extraction, but these processes are generally more time consuming and have higher material costs – thus limiting their use in high-throughput workflows. For example, a common method for extracting from capsule filter lysate is to collect the 2 mL of lysate into a tube. Then, one centrifuges the tube for 1 hour at 20000 *g* to pellet the DNA, adds 800 µL lysis buffer + proteinase K to resuspend the pellet, and carries the 800 µL of solution through standard extraction steps [23]. The main bottleneck of this method is that high-volume (> 2 mL tubes) benchtop centrifuges generally have no more than a 48-sample capacity, so this step adds at least two hours per plate of 96 samples when using just one centrifuge. Additionally, extraction efficiency may be lower with a centrifugation step, because the relatively lower molecular weight of extracellular DNA may make it less likely to pellet during centrifugation, potentially lowering the DNA yield or biasing DNA extraction towards cell- or organelle-bound DNA. To precipitate unpelleted DNA, ethanol and sodium acetate can also be added after centrifugation and stored overnight at −20 °C [53], thus adding more sample handling and processing time to the procedure. Alternatively, the amount of lysate in a filter can be reduced by preserving capsule filters in the field with ethanol instead of a lysis-preservation buffer. Since only a small amount of DNA is expected to be pulled into ethanol off a filter, the ethanol is then discarded in the laboratory, and the filter is air-dried for at least one hour to remove residual ethanol. As with the previous protocol, 800 µL of ATL buffer + proteinase K is added, but to the capsule filter itself. The capsule filter is then shaken and incubated for 3 hours, and then the full 800 µL of solution is used in subsequent, standard extraction steps [23,54].

Another method to increase the volume of lysate extracted would be to increase binding steps. With the magnetic bead binding approach used here with liquid handling robots that only have 8 channels of pipettes, additional binding steps of 350 µL lysate each can be repeated beyond the two performed in the present study, which would take 1.5 hours per additional binding step of 96 samples and ~US$4 per sample in reagents with our configuration. Using a 96-channel head, each binding step would only take 45 minutes. For column-based extraction kits, additional binding steps would be performed as iterative loading of lysate to spin columns, though spin columns generally can bind only up to 100 µg of DNA before saturation. Finally, multiple aliquots of lysate could be extracted separately until all lysate is extracted. Extracts could then be analyzed separately or eluted to smaller volumes and pooled into a standard final volume. However, DNA yield can decrease by over 5% for each 20 µL reduction in elution volume below 100 µL for common DNA kits (e.g., one commercially available spin-column DNA extraction kit recommends a minimum elution volume of 35 µL, which yields approximately 20% less DNA than a 100 µL elution [55]). Given the many ways to increase laboratory effort, further controlled experiments are needed to test the extent to which greater extraction efficiency alone affects species detections, and its trade-offs with additional time and costs. Since our results suggest that the higher proportion of lysate that was extracted from disc filters may have contributed to the detection of more species, additional extraction effort of capsule filters may be worth the additional processing cost if the alternative is analyzing more field samples.

In contrast to marine vertebrates, we found little difference between filter types in terms of general eukaryote (metazoans and phytoplankton, 18S rRNA) species detection per sample. Simulations that varied initial DNA template concentration did not reproduce the observed similarity between filtration approaches, indicating that higher template concentration alone may not explain these patterns. Additional simulations varying skewness, PCR amplification uniformity, sequencing depth, and stochastic capture by the filter also did not reproduce the observed patterns. Taken together, these results suggest that the observed similarity in general eukaryote species accumulation between filtration approaches is not fully explained by the factors tested here and may reflect additional contributing mechanisms.

Disc and capsule filtration approaches were comparable on a per sample basis for 18S rRNA richness, which means that capsule filters detected more species with 18S rRNA per effective volume filtered than disc filters. Consistent with this similarity in richness, disc and capsule filters detected largely overlapping eukaryote assemblages, with relatively small compositional differences driven by dispersion rather than shifts in group centroids. One possible explanation for this difference in detection per effective volume is that the capsule filters had a slightly smaller pore size rating than the disc membranes, which could lead to the capture of smaller-bodied organisms. However, the pore size of a nylon filter is defined by its maximum pore size, so a 1-µm pore size nylon filter still has many smaller pores that could trap the same small-bodied organisms. Additionally, as a filter clogs, the maximum and mean pore size necessarily becomes smaller. Filter pore size and membrane type have been shown to have anywhere from small (e.g., no difference in perch detection rate among polyethersulfone [PES], cellulose nitrate, and glass fiber filters [23]) to large (e.g., cellulose nitrate filters yielding up to 3 times the richness of glass fiber [56]) effects on species detection. Since membrane type and pore size were two of the major unaccounted for differences between disc and capsule filters in our study, further work could hold pore size and membrane type constant between the two filtration approaches to determine whether this is a major driver of observed 18S rRNA patterns. However, regardless of the mechanism, since there was no strong difference in 18S rRNA species detection between disc or capsule filters on a per sample basis, disc-based filtration approaches would be the most cost-effective option under the conditions tested here based on the difference in filter cost alone: US$1 versus US$15 per unit.

In addition to comparing disc versus capsule filtration approaches, we tested the benefit of field versus laboratory replicates when using 12S rRNA MiFishU primers. We observed similar detection rates whether a given amount of PCR and sequencing effort was applied within a single field sample or spread across multiple field samples, with only one to two more species per sample detected with field samples over PCR replicates. Thus, additional field samples spaced from 15 m – 150 m apart may not be more beneficial along the short intertidal transect studied here than increasing the number of PCR replicates per sample. Nevertheless, other studies have documented strong spatial structuring of eDNA-based assemblages in coastal regions at finer spatial scales than the 150-m transect we sampled, suggesting that the findings of this study may not be directly transferable to systems with higher spatial heterogeneity, where increasing independent field samples may be essential for representative detection. In a California rocky intertidal habitat similar to the one examined here, general eukaryote assemblages based on ESVs from triplicate field replicates were different among three sites spaced 40 m apart [57]. Across a gradient of marine habitats associated with a kelp forest ecosystem in California, eDNA-based marine vertebrate assemblages differed by habitat when separated by 60 m [58]. In contrast, along our 150-m rocky intertidal transect, marine vertebrate DNA did not differ strongly by site, indicating that the DNA was homogeneously distributed beyond the scale of the water we filtered for an individual sample. Altogether, our results suggest that PCR replicates can be a more efficient and cost-effective option than field replicates for detecting richness at a single intertidal site, but there is currently no *a priori* method to determine spatial gradients in eDNA – and thus the variation among field samples – without the sampling itself.

In addition to showing that independently sequencing multiple PCR replicates effectively characterizes richness along a rocky intertidal field transect, we found that pooling the PCR replicates after amplification and then sequencing this single pooled sample captured similar richness as did all 10 independent PCR replicates combined – with or without controlling for sequencing depth. A single pooled sample detected double the richness as a single independent PCR replicate. Like ours, several studies have shown that increasing the number of independent PCR replicates yields greater diversity detection beyond the benefits of additional sequencing depth [59,60]. Meanwhile, other studies have pooled anywhere from 3 [61] to 12 [62] first round PCR replicates to reduce variability and increase species detection. For instance, [63] found that an 8-replicate pool detected 2.5 times more fish OTUs and up to half the Bray-Curtis dissimilarity of a single replicate.

To our knowledge, only the present study and [64] have directly compared richness estimated from pools of first-round PCR replicates to that from the same number of independent PCR replicates sequenced separately. In contrast to our findings, [64] reported that in 20 freshwater stream eDNA samples, 12 independent PCR replicates detected on average 1.5 times more vertebrate species than pools of the same replicates, and that a single pool recovered richness comparable to that of a single replicate. Their generalized linear mixed models further suggested that pooling disproportionately enhances the detection of taxa common across replicates while diluting signal from rare species. The reasons for our contrasting outcomes of pooling PCR replicates remain unclear. Possible explanations include ecological differences between marine and freshwater systems, differences in post-bioinformatics filtering criteria (in our study, we removed observations with < 8 reads, while [64] removed observations with < 0.1% relative abundance), and variation in how evenly species are distributed within (relative abundance) and across (prevalence) samples. These results highlight that the extent to which pooling PCR replicates can reliably capture richness is still poorly understood and may depend on both methodological choices and underlying ecological context.

Further work is needed to determine if there is a saturation point at which pooling PCR products does not capture similar diversity as independently sequenced, constituent PCR replicates, since the relative concentration of each PCR replicate in the pooled sample decreases with each additional replicate when a fixed volume of the pooled sample is used in second round PCR. Likely, the effectiveness of pooling also interacts with the sequencing depth achieved, with more shallowly sequenced pools leading to lower than expected diversity [65]. In addition, the heterogeneity of template across PCR replicates may affect the efficacy of pooling, as some studies have shown that as few as 8 [66] or 20 [26] independent replicates are sufficient to reach asymptotic species accumulation. Meanwhile, [34] found that 24 replicates were still insufficient for detecting maximum diversity for two different primer sets. Likewise, in our study, detecting 95% of asymptotic richness per field sample required over 50 independent PCR replicates for both capsules and discs – more than the 33 PCR replicates possible from a single 100 µL eluate of extracted DNA. These results suggest that a single pool of all extracted DNA in a sample could still provide new information in samples with high variability among PCR replicates. Therefore, despite remaining uncertainties about pooling saturation or universality of the effectiveness in pooling, pooling PCR replicates offers yet another opportunity to reduce laboratory effort and cost with no detectable difference on richness compared to independently processed replicates in this dataset.

Given the greater richness detected with disc filters than capsule filters and the equivalence of PCR replicates within and among samples, an efficient eDNA sampling approach for maximizing richness detection from our intertidal site at Point Conception is to pool as many first round PCR replicates from a single disc filter as possible, sequencing the pooled sample, and then sequencing subsequent PCR replicate pools from additional field samples. Increasing the number of PCR replicates from an existing DNA extract avoids repeated extraction and field sampling steps and therefore reduces laboratory effort and costs relative to collecting and processing additional field samples. The magnitude of these savings will depend on laboratory workflow, project scale, batch size, sequencing arrangements, and other provider- or laboratory-specific factors. Given that 3 µL of 100 µL eluted DNA extract is used for a single PCR reaction, each field sample can provide up to 33 PCR replicates. Yet, we predicted that regional species pools would not be detected until at least 700 (12S rRNA) and 100 (18S rRNA) samples (field samples or PCR replicates) were analyzed. Thus, at least 20 field samples would still need to be fully analyzed (33 PCR replicates each) to capture the entire regional species pool.

In this study, laboratory effort – including hands-on personnel time and the number of extraction steps – was held constant across two filtration approaches to enable a standardized cost comparison. Additional laboratory processing of capsule filters could lead to a lower cost per species detected. All the lysate from capsule filters could be extracted into a single extraction aliquot using either lysate concentration (centrifugation and/or precipitation), pooling separately extracted elutions, or running more binding steps. The extent to which this additional extraction effort would scale with species detection remains an open question and could be a topic for research.

Pooling PCR replicates also has the potential to reduce costs since many of the costly PCR, indexing, and sequencing steps are the same for multiple pooled PCR replicates as just one. Pooling PCR replicates would not allow for accumulation curves to be generated or estimates of asymptotic richness from PCR replicates, but it would reduce costs. However, more controlled research would be necessary to compare species detections with pooled PCR replicates versus independent PCR replicates at scale.

Analyzing eDNA with metabarcoding detected reasonable assemblages of fishes, marine mammals, marine invertebrates, and phytoplankton, with many of the provisionally assigned species known to occur in coastal California. Our consensus species assignment algorithm emphasized assigning ESVs to an internally consistent species-level taxonomic rank rather than resolving definitive species identities. As with any eDNA study, accurate species-level taxonomic assignments are limited by reference database completeness and amplicon resolution. For well-studied metazoans like marine vertebrates, the top reference matches to ESVs can be compared to known distributions to prioritize hits that represent likely species versus closely related but unlikely species. Yet, for groups with sparse reference databases and poorly known distributions, accurate species identifications are more difficult, so names are often necessarily provisional at more specific taxonomic ranks. For instance, the California intertidal study by [57] found that only 72% of consensus taxa from a cytochrome c oxidase subunit I (COI) primer set were actually from the sample region. In our case, 96% of the 12S rRNA species were plausible locally when compared to a list of known species from the region (S3 Table). The three non-local species were common aquarium fishes, each detected in only one or two samples. Given the high plausibility of the resulting species assignments, our approach appears effective for estimating species richness while generating biologically reasonable, albeit provisional, species-level assignments.

## Conclusions

This study demonstrates that cost-effective eDNA sampling and laboratory methods can match or even outperform more expensive approaches for species detections when laboratory effort is held constant. When extracting the same volume of lysate from two different filter types, we found that the filtration approach using lower-cost 5-cm^2^ disc filters detected more marine intertidal vertebrate species (12S rRNA) than the approach using larger, more expensive 10-cm^2^ capsule filters. Modeling indicated that this pattern is consistent with extracting a higher proportion of lysate from disc filters, increasing the effective water volume processed, although filtration membrane material, pore size, and lysis buffer chemistry were not isolated here. Thus, these findings reflect comparisons of complete filtration-extraction workflows under standardized laboratory effort, and differences between filtration approaches cannot be attributed to any single component. More research would be required to understand any independent roles of these factors and under what workflows capsule filters can provide equivalent results to the smaller disc filters for rare targets. For general eukaryote detection (18S rRNA), disc and capsule filters performed similarly, suggesting that filtration approach may have less influence on detecting diversity of broad taxonomic groups under the conditions tested here. Thus, for these groups, eDNA programs might prioritize methods and supplies that reduce cost and effort during sampling and processing. We also found that lower-cost technical PCR replicates detected as many species as more expensive field samples along a 150-m intertidal transect, indicating that more comprehensively analyzing a single sample can reduce costs without sacrificing detection, although identifying which ecosystems this would apply to more generally remains to be seen. Furthermore, pooling PCR replicates after amplification yielded equivalent richness to sequencing individual PCR replicates, indicating that pooling PCR replicates can be a cost-effective approach for richness-based analyses when PCR effort-response curves are not required. These findings highlight that some sample acquisition and processing strategies can lead to greater species detection while minimizing cost and hands-on time.

## Supporting information

S1 Table Metadata and sequencing summary statistics for January 2024 sampling. (XLSX)

S2 Table Metadata and sequencing summary statistics for May 2024 sampling. (XLSX)

S3 Table Provisional taxonomic assignments and read counts for 12S rRNA January 2024 samples. The sequence corresponds to the representative ESV for each consensus species assignment.
(XLSX)

S4 Table Provisional taxonomic assignments and read counts for 18S rRNA January 2024 samples. The sequence corresponds to the representative ESV for each consensus species assignment.
(XLSX)

S5 Table Provisional taxonomic assignments and read counts for 12S rRNA May 2024 samples. The sequence corresponds to the representative ESV for each consensus species assignment.
(XLSX)
